# Bridging the Gap Between Immediate Surgical and Definitive Oral Rehabilitation With Interim Obturators: A Case Report

**DOI:** 10.7759/cureus.79103

**Published:** 2025-02-16

**Authors:** Saumil C Sampat, Sohan S Raje, Jyoti B Nadgere, Janani Iyer, Prachiti M Terni

**Affiliations:** 1 Department of Prosthodontics, Mahatma Gandhi Mission's Dental College &amp; Hospital, Navi Mumbai, IND

**Keywords:** definitive obturator, dental, hemimaxillectomy, human oral squamous cell carcinoma (oscc), hypernasal speech, interim, obturator, radiation, rehabilitation, surgical obturator

## Abstract

Maxillectomy can result in various functional deficits for the patient, such as hypernasal speech, difficulty in deglutition, fistula formation, and loss of support for cheeks and lips. Surgical reconstruction might be the ideal treatment for rehabilitation of maxillectomy defects, but it is seldom done, particularly if the defect is significant and the tissues are exposed to radiotherapy. Since a definite obturator is deferred till complete healing of the surgical site has occurred, an interim obturator helps in achieving normalcy. Apart from restoring the functions of speech and deglutition, the interim obturator has a great psychological benefit to the patient in regaining social acceptance. In this case report, we describe how our patients benefitted from an interdisciplinary team approach with the help of interim obturators till the surgical site attained stability for the fabrication of definite obturators, thereby improving their overall quality of life.

## Introduction

Maxillofacial deformities can be congenital, acquired, or developmental based on their origin [1-5]. Acquired defects are those that are caused by surgical intervention to eliminate the cause of the disease process or from trauma, causing significant alterations in the orofacial complex [6]. Oral cancers are commonly implicated factors for acquired maxillary defects. Cancers of the lip and oral cavity rank 13th, with the two most important etiological factors being smoking and chewing tobacco [7]. Histopathological subtypes of malignancies commonly found at the maxillary sinus include squamous cell carcinoma, adenocarcinoma, adenoid cystic carcinoma, salivary gland carcinoma, or malignant melanoma. Treatment consists mainly of surgery and/or radiotherapy, and planning for either is complicated by the complex anatomy of the region with proximity to vital structures such as the brain, eyes, and cranial nerves [8-10]. Surgery and/or radiotherapy may lead to changes in the biomechanics of the orofacial complex, resulting in changes in occlusion, vertical dimension, and significant deviation to the resected side with aberrant mandibular closure patterns. A maxillectomy can also result in hypernasal speech and fluid leakage along with the previously stated potentially disabling issues. While surgical reconstruction of the defect remains the ideal choice, it may not often be enough to close large defects for which prosthetic rehabilitation is required. The prosthetic rehabilitation for maxillectomy patients aims at the separation of the oral and nasal cavities to allow adequate deglutition and articulation in order to restore the mid-facial contour and to provide acceptable results. This, in turn, helps to improve the quality of life of the patient [11].

The obturator is a maxillofacial prosthesis used to close and maintain the integrity of the oral and nasal compartments that are altered because of a congenital, acquired, or developmental disease [12]. Based on the timing of prosthodontic intervention, the treatment phases can be divided into the pre-operative phase, which involves the fabrication of an immediate surgical obturator, and the post-operative phase, utilizing an interim obturator and/or a definitive obturator. The immediate surgical obturator is used to restore the continuity of the hard palate and contiguous structures immediately after surgery, while the interim obturator is made several weeks or months following surgical resection of a portion of one or both maxillae. It may include the replacement of teeth in the defect area. A definitive obturator artificially replaces part or all of the maxilla and the associated teeth lost due to surgery or trauma once the tissues have completely healed and become stable [13].

This case report highlights the importance of the interim obturator, which helps to eliminate oro-antral communication and hypernasal speech and restore oral masticatory function, thus contributing to the overall psychological well-being of the patient. The authors also give a detailed account of the fabrication of the prosthesis for the individual cases.

## Case presentation

Case 1

In the first case, a 41-year-old male individual reported to the Department of Prosthodontics, Mahatma Gandhi Mission’s Dental College & Hospital, Kamothe, Navi Mumbai, Maharashtra, India, with a chief complaint of being unable to speak and eat post maxillectomy of the right side. The patient was operated on for a nonhealing ulcer on the right maxillary buccal mucosa. The histopathological report suggested an invasive, moderately differentiated squamous cell carcinoma, conventional (keratinizing) of the right retromolar area, maxillary vestibule of mouth, upper gingiva and hard palate involving the upper alveolar bone, and maxillary sinus mucosa with metastases, to multiple ipsilateral neck lymph nodes. Pathologic stage classification of the lesion was pT4aN2b [14].

**Figure 1:**
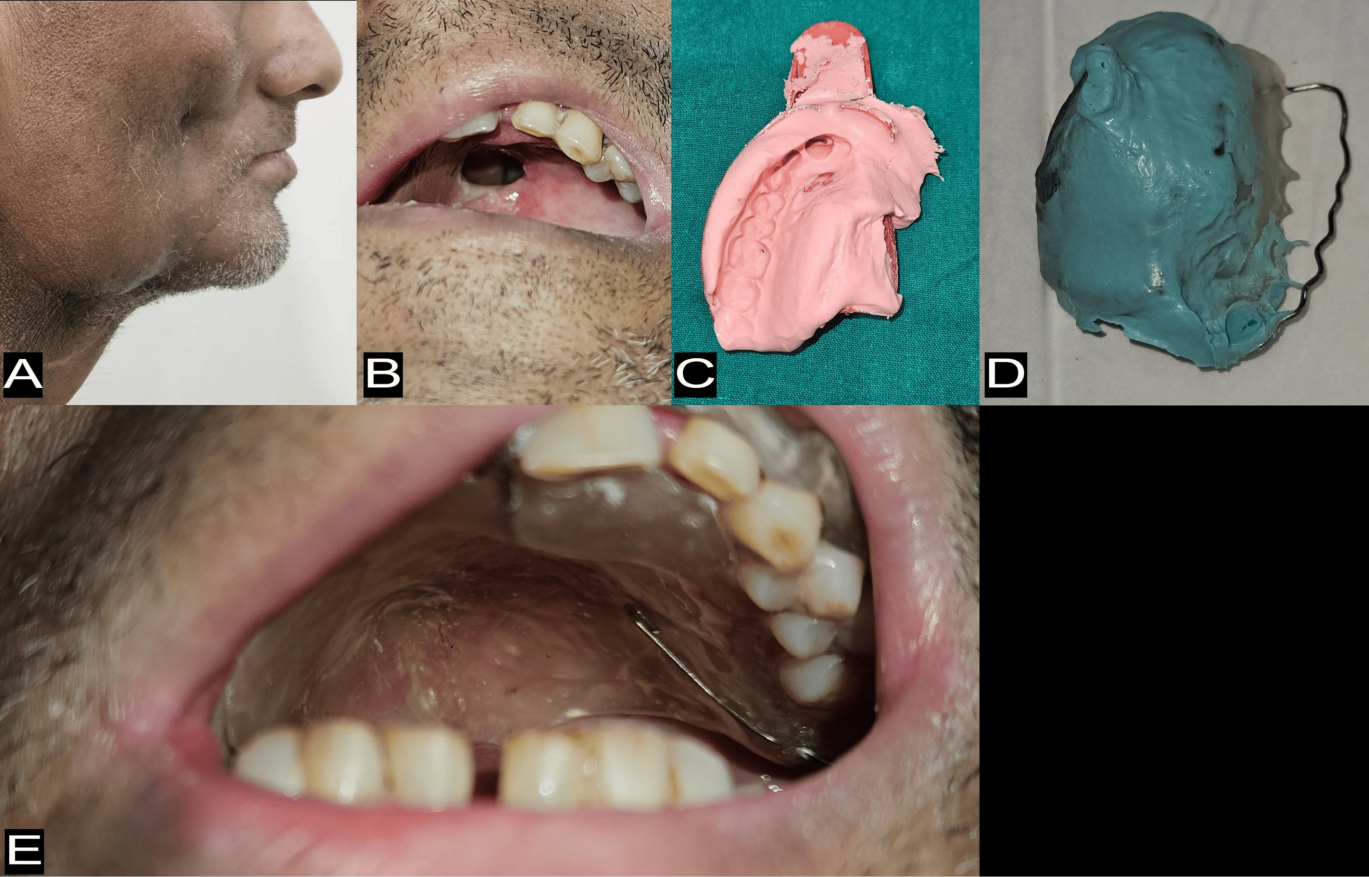
Extraoral view, intraoral view and impressions for fabrication of interim obturator for case 1. (A) Right lateral extraoral view showing radiotherapy marks and tethering of overlying skin; (B) Intraoral view of the defect; (C) Primary impression of the defect made using modified dentulous plastic stock tray and irreversible hydrocolloid; (D) Final impression of the defect made using custom tray retained with wrought wire continuous circumferential clasp; (E) Intraoral view of interim obturator in place. Additional retention was provided by completely encasing the clasp assembly in clear acrylic resin to engage teeth undercuts.

The squamous cell carcinoma was unifocal in nature and extended to the right retromolar area, vestibule, upper gingiva, and hard palate of the mouth. For the treatment, the patient had to undergo a right infrastructure maxillectomy with postoperative radiotherapy (Figure 1A). However, the patient reported six months later, unable to drink and eat as there was an oro-antral communication due to contraction of the flap. Since the tissues were unstable at the time of reporting the patient to the institute, an interim obturator was decided upon (Figure 1B). A primary impression of the maxillary arch was made using a modified dentulous plastic stock tray and irreversible hydrocolloid impression material (Impreceed, GC Corporation, Tokyo, Japan) (Figure 1C). A primary cast was obtained, and a specialized custom tray was fabricated using cold-cured acrylic resin (DPI® RR Cold Cure Acrylic Repair Material, The Bombay Burmah Trading Corporation Limited, Mumbai, India), with a 19-gauge wrought wire continuous circumferential clasp adapted around the dentulous portion. In the next appointment, the defect area was recorded using low-fusing green stick compound (DPI® Pinnacle Tracing Sticks, The Bombay Burmah Trading Corporation Limited, Mumbai, India), and a final wash impression was made with medium body viscosity polyvinyl siloxane impression material (Avue® Gum Mono, Dental Avenue, Gurgaon, India) and the custom tray (Figure 1D). An altered cast technique was used to fabricate the final/master cast. A clear interim obturator was made of cold-cured acrylic resin (DPI® RR Cold Cure Acrylic Repair Material). The opening in the patient’s mouth was completely closed with the interim obturator. The patient had instant relief and was able to eat and drink without regurgitating (Figure 1E).

Case 2

**Figure 2:**
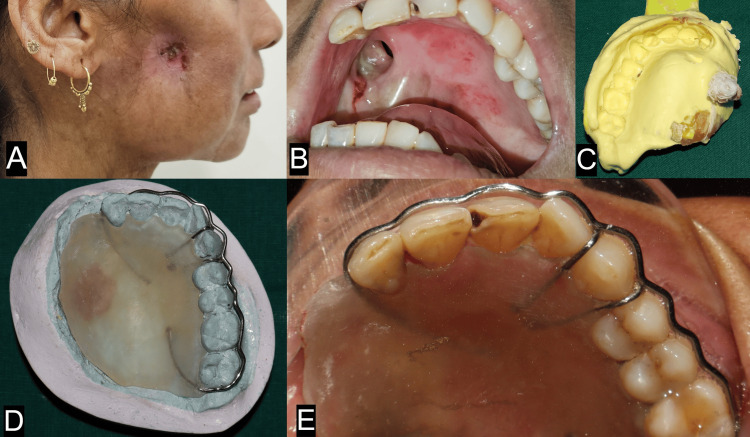
Extraoral view, intraoral view and impression for fabrication of interim obtuartor for case 2. (A) Right lateral extraoral view showing fistula formation at cheek; (B) Intraoral view showing oro-antral communication and purulent discharge from the defect; (C) Primary impression of the defect made with a modified dentulous plastic stock tray and irreversible hydrocolloid; (D) Primary cast with clasp assembly; (E) Intraoral view of interim obturator in place showing closure of the defect.

In the second case, a 42-year-old female individual presented with a chief complaint of being unable to eat and drink with nasal regurgitation and pus discharge from the right cheek. Previous medical history revealed recurrent carcinoma of the right buccal mucosa for which she had undergone a partial maxillectomy two years ago (Figure 2A). However, there was a fistula as well as an intraoral communication present post-surgery and healing. The patient’s mouth opening was about 17 mm, and there was perceivable contracture of the operated side tissues as well (Figure 2B). A primary impression was made using a modified dentulous plastic stock tray and irreversible hydrocolloid (Tropicalgin, Zhermack SpA, Badia Polesine, Italy) (Figure 2C). A primary cast was subsequently poured, and modeling wax was used to block out the undercuts. Ball end clasps and a continuous circumferential clasp were added to the design of the obturator for maximum retention. Self-cured clear acrylic resin (DPI® RR Cold Cure Acrylic Repair Material) was used to make the plate (Figure 2D). Adequate retention was present in the interim obturator, and the patient reported improvement in mastication and reduction in the hypernasality of the voice (Figure 2E).

## Discussion

The lining of the oral mucosa, when exposed to tobacco in a susceptible individual, can become the site of benign, pre-malignant, and malignant lesions. Treatment depends on how early the tumor is detected, its location, type, and extent of spread. Advanced oral squamous cell carcinoma (OSCC) has a 20% five-year survival rate [15]. An established and popular treatment for maxillofacial cancer is surgical resection. Post-surgical resection of maxillary cancers, the patient encounters several deficits in speech, mastication, and deglutition due to oro-antral communication. The resulting defects, if small, can be closed with surgical reconstruction using microvascularized free flaps; however, prosthetic reconstruction is often required since reconstructive surgery alone is frequently insufficient to fix the deficiencies, particularly when the defect is significant [13]. Post-operative radiotherapy can also cause significant wound contraction and flap dehiscence, thus compromising the stability of the tissues. The separation between the oral and nasal cavities to allow adequate deglutition and articulation, with the restoration of facial contours, is the primary of any surgical or prosthetic rehabilitation of maxillectomy defects.

The earliest documented evidence of rehabilitation of maxillary defects with an obturator dates back to the 16th century when Ambroise Paré popularized the concept [16]. Prosthetic rehabilitation can be divided into three phases (immediate/surgical obturator, transient/interim obturator, and definitive obturator) based on the timing of intervention. The primary purpose of the immediate/surgical obturator is to provide support for the graft and surgical pack, thus isolating the surgical site and reducing the chances of infection [13]. In addition, the surgical obturator also reduces the psychosocial impact of the surgery and enables the patient in speech, mastication, and swallowing. The fabrication of an immediate surgical obturator requires a pre-operative cast, thus necessitating an interdisciplinary approach between the surgical team and the maxillofacial prosthodontist to determine the extent of resection. Interim obturators are normally placed seven to 10 days after surgery and can be expected to undergo several modifications to compensate for dimensional changes that occur in the tissues due to scar contracture and wound reorganization.

While immediate and interim obturators are made of cold cure acrylic resins for ease of fabrication and modification, definitive obturators incorporate rigid cobalt chromium frameworks with heat cure acrylic poly methyl methacrylate resins, which are difficult to adjust. Thus, it recommended that patients be sufficiently accommodated to the interim prosthesis to maximize prosthetic success during the definitive phase. The design of definitive obturators is based on the classification of the defect and principles of removable prosthodontics, such as retention, stability, support, broad stress distribution, and cross-arch stabilization must be adhered to [17-19]. Financial limitations, ease of access to healthcare, recurrence of tumors, and radiotherapy are some of the factors that preclude the fabrication of a definite obturator. Nevertheless, the overall quality of life (QOL) and speech of the patients show significant improvement with the use of surgical obturators, immediate obturators, and definitive obturators in that order [20].

## Conclusions

Maxillectomy defects present unique challenges for the maxillofacial prosthodontist, and while the timeline of a definitive obturator is crucial, the interim obturator plays a pivotal role in stabilizing oro-facial tissues and function. While these two cases clearly demonstrate the benefits of interim obturators, further research with larger sample sizes and long-term follow-up is needed to substantiate these findings. Nevertheless, this case report serves to highlight the importance of the interim obturator in facilitating ora-antral separation, reducing hypernasal speech, improving mastication, and, thus, the overall psychosocial impact of a potentially debilitating surgical procedure.

## References

[1] Plank DM, Weinberg B, Chalian VA (1981). Evaluation of speech following prosthetic obturation of surgically acquired maxillary defects. J Prosthet Dent.

[2] Wheeler RL, Logemann JA, Morton SR (1980). Maxillary reshaping prosthesis: effectiveness in improving speech and swallowing of post-surgical oral cancer patients. J Prosthet Dent.

[3] Sawhney A, Dwivedi H, Singh S, Dhar S, Gupta S, Choudhary P (2016). Hollow bulb obturator-a simplified approach (case report). Arch Dent Med Res.

[4] Deogade SC, Mantri SS, Naitam D, Dube G, Gupta P, Dewangan A (2013). A direct investment method of closed two-piece hollow bulb obturator. Case Rep Dent.

[5] McAndrews KS, Rothenberger S, Minsley GE (1998). An innovative investment method for the fabrication of a closed hollow obturator prosthesis. J Prosthet Dent.

[6] Rieger J, Wolfaardt J, Seikaly H, Jha N (2002). Speech outcomes in patients rehabilitated with maxillary obturator prostheses after maxillectomy: A prospective study. Int J Prosthodont.

[7] Hernández-Morales A, González-López BS, Scougall-Vilchis RJ (2023). Lip and oral cavity cancer incidence and mortality rates associated with smoking and chewing tobacco use and the Human Development Index in 172 countries worldwide: an ecological study 2019-2020. Healthcare (Basel).

[8] Kreeft AM, Krap M, Wismeijer D (2012). Oral function after maxillectomy and reconstruction with an obturator. Int J Oral Maxillofac Surg.

[9] Itami J, Uno T, Aruga M, Ode S (1998). Squamous cell carcinoma of the maxillary sinus treated with radiation therapy and conservative surgery. Cancer.

[10] Madison Michael L 2nd, Sorenson JM, Samant S, Robertson JH (2005). The treatment of advanced sinonasal malignancies with pre-operative intra-arterial cisplatin and concurrent radiation. J Neurooncol.

[11] Kornblith AB, Zlotolow IM, Gooen J (1996). Quality of life of maxillectomy patients using an obturator prosthesis. Head Neck.

[12] Punjabi AR, Mistry G, Shetty O, Rathod A (2019). Maxillary hollow-bulb obturator: a paradigm shift. J Indian Prosthodont Soc.

[13] Dalkiz M, Dalkiz AS (2018). The effect of immediate obturator reconstruction after radical maxillary resections on speech and other functions. Dent J (Basel).

[14] Amin MB, Edge SB, Greene FL (2017). AJCC cancer staging manual, 8th edition. https://link.springer.com/book/9783319406176.

[15] Ford PJ, Rich AM (2021). Tobacco use and oral health. Addiction.

[16] Bulbulian AH (1965). Maxillofacial prosthetics: evolution and practical application in patient rehabilitation. J Prosthet Dent.

[17] Desjardins RP (1978). Obturator prosthesis design for acquired maxillary defects. J Prosthet Dent.

[18] Aramany MA (1978). Basic principles of obturator design for partially edentulous patients. Part I: classification. J Prosthet Dent.

[19] Aramany MA (1978). Basic principles of obturator design for partially edentulous patients. Part II: design principles. J Prosthet Dent.

[20] Dholam KP, Bachher G, Gurav SV (2020). Changes in the quality of life and acoustic speech parameters of patients in various stages of prosthetic rehabilitation with an obturator after maxillectomy. J Prosthet Dent.

